# Basaloid Cell Hyperplasia Overlying Dermatofibroma

**DOI:** 10.3390/dermatopathology12040036

**Published:** 2025-10-10

**Authors:** Pablo Izarra, Marwa Zohdy, Helmut Beltraminelli, Laurence Feldmeyer

**Affiliations:** 1 Department of Dermatology, Inselspital, Bern University Hospital, University of Bern, 3010 Bern, Switzerland; pablo.izarracastanos@students.unibe.ch; 2 Department of Dermatology, Andrology and STDs, Mansoura University Hospitals, Mansoura University, Mansoura 35516, Egypt; marwazohdy@mans.edu.eg; 3 Department of Dermatology, Ente Ospedaliero Cantonale (EOC), Università della Svizzera Italiana (USI), 6500 Bellinzona, Switzerland; helmut.beltraminelli@eoc.ch; 4 Department of Dermatology, University Teaching and Research Hospital of the University of Lucerne, 6000 Lucerne 16, Switzerland

**Keywords:** basaloid cell hyperplasia, dermatofibroma, basal cell carcinoma, Ber-EP4, CK-20, Ki-67

## Abstract

Dermatofibromas (DFs) are benign neoplasms of the dermis typically found on the extremities of young adults. In approximately 3–5% of cases, basaloid cell hyperplasia (BCH) is observed overlying DFs. BCH is characterized by the proliferation of basaloid cells within the epidermis. BCH and superficial basal cell carcinoma (BCC) share many histological features, making their differentiation challenging. It is therefore unclear if the proliferation of basaloid cells in DFs represents an inductive process or, conversely, a malignant transformation indicative of BCC. The primary objective of our study was to determine whether BCH can be distinguished from superficial BCC using histology and immunhistological techniques. The histological and immunohistochemical characteristics of 43 DF samples with overlying BCH revealed significant similarities in staining patterns with those of superficial BCC described in the literature. These findings point to the need for innovative methods, such as molecular techniques, to refine diagnostic accuracy.

## 1. Introduction

Dermatofibromas (DFs) are common benign dermal neoplasms typically found on the extremities of young to middle-aged women, particularly between 20 and 50 years of age. Clinically, they present as firm, slow-growing papules or nodules, often less than 1 cm in diameter, although larger variants—sometimes referred to as giant dermatofibromas—have been documented in the literature. The lesions are generally asymptomatic but may be pruritic or tender upon palpation. One of the classic clinical signs is the “dimple sign,” where lateral compression of the lesion results in a central depression due to dermal tethering, a feature highly suggestive of DF. Epidemiological studies report a clear female predominance, with a female-to-male ratio of approximately 2:1. A history of local trauma—such as insect bites, superficial injuries, and puncture wounds—is present in up to 20% of cases, supporting the theory of a reactive or post-inflammatory etiology in at least a subset of lesions [1,2,3].

Histologically, DFs are characterized by a proliferation of spindle-shaped fibroblast-like cells arranged in a storiform pattern within the dermis, often accompanied by peripheral collagen trapping, and a variable inflammatory infiltrate. A particularly notable histological feature is the overlying reactive epidermal hyperplasia, which may include marked acanthosis, hyperkeratosis, elongation of the rete ridges, and basal layer hyperpigmentation—collectively referred to as the “dirty feet sign” [1]. These changes are not merely incidental findings but occur in a substantial proportion of cases. For instance, Senel et al. report hyperkeratosis in up to 86% of DF cases, acanthosis in 81%, and basal pigmentation in 57%, emphasizing the frequency and diagnostic relevance of epidermal changes in these lesions [3].

In approximately 2–5% of cases, a more distinct proliferative phenomenon occurs within the basal epidermis—known as basaloid cell hyperplasia (BCH) [4]. BCH is defined by the expansion of small, darkly staining basaloid keratinocytes arranged in nests or cords, which can mimic the histological appearance of superficial basal cell carcinoma (BCC) [5]. Similar basaloid proliferations have also been reported in other benign cutaneous lesions, including hamartomas, cutaneous myxomas, and seborrheic keratoses. Although less extensively studied than in dermatofibromas, these proliferations appear histologically indistinguishable from those in DF [6,7,8]. Such findings highlight that BCH can arise across a spectrum of benign conditions, thereby compounding the risk of confusion with basal cell carcinoma. The resulting diagnostic uncertainty is considerable, as both entities may display peripheral palisading, basaloid lobules, and epidermal budding. Given the vastly different biological behavior and therapeutic implications of BCC versus BCH, accurate diagnosis is of paramount importance. Misinterpreting a benign hyperplastic change as malignant can lead to unnecessary surgical interventions, psychological distress for the patient, and unwarranted healthcare cost.

The biological basis for the development of BCH over DF remains a subject of ongoing investigation. One of the leading hypotheses involves the concept of induction, whereby mesenchymal cells within the DF exert a paracrine effect on the overlying epidermis and adnexal structures. This mesenchymal–epithelial interaction is thought to be mediated by the secretion of growth factors and cytokines, including Epidermal Growth Factor (EGF) and its receptor (EGFR), leading to hyperplastic and occasionally basaloid proliferation of keratinocytes [9]. These inductive changes can simulate various other entities histologically, including seborrheic keratosis, actinic keratosis, and superficial BCC [9,10,11].

Additional studies suggest that upregulation of stem cell factor (SCF) and c-Kit (CD117) signaling within DF may also contribute to melanocytic hyperplasia in the basal layer, further complicating the histological landscape and leading to potential confusion with melanocytic lesions such as junctional nevi or melanoma in situ [10]. Moreover, DFs are capable of inducing adnexal structures, including follicular and sebaceous elements, in what has been described as follicular and mantle induction, respectively. These forms of differentiation occur more frequently than previously appreciated and may manifest as follicular germinative cells forming primitive mesenchymal papillae or as mature sebaceous lobules connecting to the epidermis. Histologically, these patterns can resemble benign adnexal neoplasms such as trichoblastoma, trichoepithelioma, or reticulated acanthoma with sebaceous differentiation [11].

The controversy surrounding BCH is centered on its biological nature. Is it a reactive epidermal phenomenon secondary to the stroma of DF, or does it represent an early stage of BCC, i.e., a neoplastic process with malignant potential? Several authors proposed that BCH is a form of benign induction with no malignant potential, citing the absence of invasive behavior or metastatic capability [12,13]. Others raised concern that BCH could represent an incipient form of BCC, particularly given its histologic similarity and, in some cases, its immunohistochemical profile [14,15]. These diverging interpretations underscore the need for a more precise diagnostic framework supported by objective molecular or immunohistochemical criteria.

Previous attempts to distinguish BCH from BCC histologically have proven challenging [5]. Therefore, adjunctive diagnostic tools such as immunohistochemistry (IHC) have been proposed to improve diagnostic confidence. Commonly used markers include Ber-EP4, which is generally positive in BCC but may also stain BCH; Ki-67, a marker of cellular proliferation; and cytokeratin 20 (CK-20), typically used to highlight Merkel cells, which are often preserved in benign lesions but lost in malignant counterparts [14,15,16]. However, the sensitivity and specificity of these markers remain under debate, and their expression patterns in BCH overlying DF have not been comprehensively evaluated in a large cohort.

The primary aim of our study was to assess whether BCH in DF can be reliably distinguished from superficial BCC using histological features in combination with a panel of immunohistochemical markers—namely Ber-EP4, Ki-67, and CK-20. By analyzing a cohort of 43 DF cases with overlying BCH, we sought to characterize their staining patterns and evaluate potential diagnostic criteria to differentiate BCH from superficial BCC. We hypothesized that although there may be considerable histologic overlap, distinct immunohistochemical profiles could help clarify the reactive versus neoplastic nature of BCH.

Our findings aim to contribute to resolving the diagnostic ambiguity surrounding BCH, improve the accuracy of histopathological evaluations, and ultimately guide more appropriate patient management.

The literature refers to this phenomenon using various terms, such as basaloid proliferation, basaloid hyperplastic epidermis, epidermal basaloid cell hyperplasia, or follicular induction over DF [12,13,14,15,16]. For consistency, we will use the term BCH throughout this study.

## 2. Materials and Methods

This study was reviewed and approved by the ethics committee of the Canton of Bern (KEK-2019-00665). Routine skin biopsies (formalin-fixed, paraffin-embedded) of patients diagnosed with DF were retrospectively examined for the presence of BCH above the lesion. The study included cases collected between 1 March 2016, and 31 December 2018. A total of 43 samples exhibiting BCH were identified.

All selected samples were stained with hematoxylin and eosin (HE) for initial morphological assessment. We evaluated following histological characteristics typically associated with BCC: the presence of basaloid lobules, peripheral palisading, basaloid cells, and peritumoral clefting. Each feature was scored as present (“1”) or absent (“0”).

Additionally, the BCC growth pattern (e.g., superficial, nodular, or infiltrative) was recorded. IHC staining was performed on all 43 samples using the BerEp4 antibody (Biosystems Switzerland AG, Muttenz). A subset of 25 randomly selected samples was also stained as a test for Ki-67 and CK-20 (both from Biosystems).

BerEp4 and CK-20 expression were scored as present (“1”) or absent (“0”). Ki-67 expression was assessed semi-quantitatively. The staining intensity was categorized as negative (“−”), weaker than the regular epidermis (“(+)”), identical to the regular epidermis (“+”), stronger than the regular epidermis (“++”), or more than double the staining of the regular epidermis (“+++”).

## 3. Results

**Figure 1:**
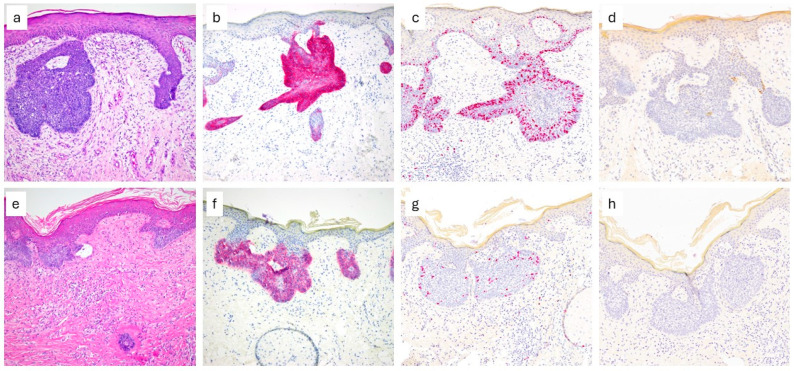
(**a**): Basaloid cell proliferation over dermatofibroma, case nr. 6, HE; (**b**): BerEp4, (**c**): Ki-67, (**d**): CK20, (**e**): basaloid cell proliferation over dermatofibroma, case nr. 7, HE; (**f**): BerEp4, (**g**): Ki-67, (**h**): CK20. All images: 100×.

All 43 DF samples with BCH exhibited a superficial growth pattern, with lobule formation and peripheral palisading. Basaloid cells were present in 40/43 samples, while peritumoral clefting was identified in 10 cases (Figure 1, Table 1). No mature hair follicles were observed, and only small sebaceous glands were identified in some cases.

Ber-EP4 staining was positive in 31/43 BCH samples (72%) and negative in 12 samples (28%). Among the 25 samples stained for Ki-67, increased proliferative activity was observed in 18 samples, with four additional cases showing more than double the staining intensity compared to the surrounding epidermis. In three cases, Ki-67 staining was simi-lar to that of the regular epidermis. CK-20 staining was positive in 12/25 samples analyzed. In nine of these positive cases, CK-20 staining was weak and limited to fewer than five cells per section (Table 1).

## 4. Discussion

Histopathological subtyping of BCC plays a pivotal role in understanding its origin, biological behavior, clinical presentation, and therapeutic approach. BCC arises from undifferentiated pluripotent epithelial germ cells located in the basal layer of the interfollicular epidermis and the bulge region of hair follicles, with tumor growth typically embedded in a fibromyxoid stroma [17,18]. Histologically, it is composed of uniform basaloid cells with hyperchromatic nuclei and scant cytoplasm, often displaying mitotic figures and apoptotic bodies. Although nuclear atypia is minimal and not correlated with clinical aggressiveness, the interaction between germinal epithelial cells and the surrounding stroma determines the formation of distinct histopathological subtypes, each associated with specific clinical behavior [17,18].

The nodular subtype remains the most prevalent, accounting for approximately 50–80% of cases and is typically characterized by peripheral palisading and a mucinous stroma containing plump cells [17]. The superficial variant, comprising 10–30% of tumors, often mimics clinically inflammatory dermatoses or in situ squamous cell carcinoma (SCC) due to its patch-like morphology and lichenoid inflammatory infiltrate [17].

Less common subtypes—such as infundibulocystic, fibroepithelial (Pinkus tumor), and micronodular BCC—present distinct histological architectures that may complicate diagnosis. Infundibulocystic BCCs resemble benign follicular adnexal tumors, while fibroepithelial BCCs are often mistaken for fibroepithelial polyps. Micronodular BCCs are clinically difficult to distinguish from superficial and nodular forms and are associated with subclinical spread and higher recurrence rates [17]. Infiltrative and morpheaform subtypes are considered more aggressive, typically showing ill-defined clinical margins and a tendency to invade deeper tissues. Histopathologically, morpheaform BCC features thin cords of basaloid cells within a densely sclerotic collagenous stroma, whereas infiltrative BCC shows narrow, angulated strands embedded in a mucinous or myxoid stroma [17]. Basosquamous carcinoma, a metatypical and rare variant combining features of BCC and SCC, accounts for less than 2% of keratinocyte carcinomas but has a significantly higher risk of metastasis (>5%) [17].

Importantly, BCC lesions can display mixed histological patterns, with nodular–micronodular combinations being the most frequent. In such cases, clinical management should be guided by the most aggressive histological component to ensure optimal therapeutic outcomes [17,18].

However, the morphological spectrum of basaloid proliferations is not exclusive to BCC. In our study, we report 43 cases of DF exhibiting BCH, a benign and underrecognized pattern that can histologically mimic BCC. This resemblance may lead to diagnostic uncertainty, especially in superficial or limited biopsy specimens. The characterization of BCH in DF is therefore essential to avoid overdiagnosis of malignancy and inappropriate treatment.

The histopathological resemblance between BCH and superficial BCC poses a significant diagnostic challenge. Both entities can display strikingly similar architectural and cytological features under routine microscopy. Superficial basaloid proliferations with a lobulated configuration and focal peripheral palisading of nuclei are frequently observed in both lesions. Furthermore, the presence of basaloid cells with small, hyperchromatic nuclei and scant cytoplasm is a shared cytological hallmark, adding to the difficulty in differentiation [16] (Table 1).

Another overlapping feature is peritumoral clefting—traditionally considered a processing artifact—which may be seen in both BCH and BCC [16,19]. These retraction spaces, located between the basaloid nests and the adjacent stroma, have been confirmed by in vivo imaging techniques such as reflectance confocal microscopy and optical coherence tomography to represent true anatomical features, often containing mucinous material. While these clefts tend to be more prominent in BCC, their presence in BCH further contributes to the histological mimicry [19].

Taken together, these similarities underscore the diagnostic limitations of conventional histology when attempting to distinguish BCH from superficial BCC, particularly in superficial shave biopsies or poorly oriented specimens. In light of these diagnostic challenges, we next examine the most relevant immunohistochemical markers reported in the literature for differentiating BCC from BCH. This includes the markers evaluated in our own cohort, Ber-EP4, Ki-67, and Cytokeratin 20 (CK20), as well as additional markers frequently highlighted in previous studies, such as metallothionein, podoplanin, and EGFR.

BerEP4 is a monoclonal antibody that targets EpCAM, an epithelial cell adhesion glycoprotein overexpressed in progenitor epithelial cells and a variety of carcinomas, including BCC [20,21]. In dermatopathology, BerEP4 is widely used for its high sensitivity and specificity in detecting neoplastic BCC cells, making it a valuable tool in differentiating BCC from other cutaneous neoplasms [21].

Its principal diagnostic application lies in distinguishing BCC from SCC, in which BerEP4 typically shows no staining. It is also employed in the evaluation of surgical margins to detect residual BCC cells, the identification of rare BCC variants—including perianal, intraoral, and metatypical forms—and the differentiation of BCC from benign adnexal tumors such as sebaceoma and microcystic adnexal carcinoma [21].

Despite its broad applicability, BerEP4 presents a critical limitation in the differential diagnosis between BCC and BCH overlying DFs. A recent study evaluating 18 immunohistochemical markers revealed that BerEP4 demonstrated similar staining patterns in BCH and in multiple BCC subtypes—including nodular, superficial, infiltrative, and morpheaform—thus undermining its reliability in this specific diagnostic context [14]. This limitation is also evident in our own study, in which 31 out of 43 samples (72%) were BerEP4 positive (Table 1), a proportion consistent with the expected immunoreactivity profile of BCC. Therefore, BerEP4 does not provide sufficient discriminatory power in cases where BCH closely mimics BCC histologically.

Ki-67 is a nuclear protein associated with cell proliferation, expressed during all active phases of the cell cycle (late G1, S, G2, and M) but absent in resting (G0) cells. As such, it serves as a well-established marker to evaluate proliferative activity and is widely used in dermatopathology for both diagnostic and prognostic purposes [22].

Studies have shown that BCC typically exhibits moderate Ki-67 expression, with average labeling indices ranging from 12–16%, depending on the subtype. Staining is frequently concentrated at the periphery of tumor nests, particularly in areas with palisading nuclei, reflecting active proliferation [13,22].

However, Ki-67 presents significant limitations when attempting to distinguish BCC from BCH associated with DFs. While some studies, such as Han et al. [13], have reported markedly lower Ki-67 indices in BEPs (~2%) compared to BCCs (~16%), suggesting a potential diagnostic discriminator, other investigations have contradicted these findings. For instance, Rossen et al. and Lindboe & Løvdal observed overlapping Ki-67 expression levels in BCH and BCC, undermining its diagnostic specificity in this context [14,22]. Moreover, and as observed in our own study, Ki-67 staining in BCCs can vary considerably among cases and even within different regions of the same tumor, further complicating its interpretation.

CK20 is a low-molecular-weight cytokeratin normally expressed in Merkel cells, as well as in epithelial cells of the gastrointestinal and urothelial tracts [23,24]. In dermatopathology, it is widely employed to identify Merkel cells, which appear as scattered intraepidermal or periadnexal elements showing strong cytoplasmic CK20 positivity [15,25].

The main diagnostic utility of CK20 lies in its ability to distinguish BCC from other basaloid proliferations, particularly trichoepithelioma and adnexal tumors. In BCC, Merkel cells are typically absent or extremely rare, and therefore CK20 staining is generally negative. In contrast, benign basaloid lesions such as trichoepitheliomas or basaloid hyperplasias frequently retain scattered CK20-positive Merkel cells, aiding in their differentiation from BCC [15,16,25,26].

However, the reliability of CK20 becomes limited when differentiating superficial BCC from BCH overlying DFs. While some studies have reported a higher density of CK20-positive Merkel cells in BCH [15], these findings are inconsistent. In several cases, BCH exhibit either a very low number or complete absence of Merkel cells, thereby reproducing the CK20-negative staining typically associated with BCC [14]. This overlap reduces the diagnostic utility of CK20 in this setting, as the absence of Merkel cells cannot be taken as definitive evidence of malignancy. Additionally, occasional CK20 positivity has been observed in rare BCC subtypes, such as infundibulocystic variants, further complicating its interpretation [16].

Beyond the markers analyzed in our cohort—Ber-EP4, Ki-67, and CK20—other immunohistochemical markers such as metallothionein (MT), podoplanin, and EGFR have also been discussed in the literature. Although not included in our study, their potential relevance in the context of basaloid lesions warrants brief consideration here.

MT is a low-molecular-weight protein involved in metal ion homeostasis and cellular protection against oxidative stress. It has been proposed as a biological marker for early carcinogenesis and shows variable expression in both normal and pathological skin [27,28].

In basal cell carcinoma BCC, MT expression has been associated with tumor aggressiveness, particularly in infiltrative or morpheaform subtypes, where it is often overexpressed. This makes MT potentially useful for distinguishing aggressive BCCs from more indolent variants [29]. However, its diagnostic value is limited in differentiating superficial BCC from basaloid cell proliferations associated with dermatofibromas. Rossen et al. reported that both superficial and nodular BCCs frequently exhibit absent or markedly reduced MT staining, mirroring the pattern observed in basaloid hyperplasia overlying DFs. Specifically, all 15 superficial BCCs and 92% of nodular BCCs included in their study were MT-negative, similar to the benign basaloid proliferations [29].

This overlapping immunoprofile diminishes the diagnostic utility of MT in this context, suggesting a possible shared metabolic or differentiation pathway between non-infiltrative BCCs and BCH. As such, MT expression alone is not a reliable marker to distinguish between these two entities.

Podoplanin is a mucin-type transmembrane glycoprotein involved in cell motility, lymphangiogenesis, and tumor invasion. Originally identified in lymphatic endothelial cells, it is widely used in pathology—particularly through the D2-40 antibody—as a marker of lymphatic vessels and as a prognostic indicator in various malignancies, including non-melanoma skin cancers [30,31].

Although podoplanin (D2-40) has been implicated in the pathogenesis of BCC, its immunohistochemical expression is highly inconsistent across studies, limiting its diagnostic reliability. In the study by Neinaa et al., no significant difference in D2-40 expression was found between BCC and seborrheic keratosis [30]. Similarly, Ishida et al. reported no correlation between podoplanin staining and specific BCC subtypes (nodular, micronodular, superficial), indicating a lack of subtype-specific expression patterns [31]. Furthermore, Tebcherani et al. demonstrated the poor performance of podoplanin in distinguishing BCC from trichoepithelioma, another benign basaloid lesion with overlapping histologic features [32].

Collectively, these findings indicate that podoplanin lacks sufficient specificity or discriminatory power to serve as a reliable marker for distinguishing BCC from BCH or other benign basaloid proliferations.

EGFR is a transmembrane receptor tyrosine kinase of the ErbB family that, upon ligand binding, activates intracellular signaling pathways regulating cell proliferation, survival, and differentiation. Under physiological conditions, EGFR plays a central role in epithelial homeostasis, including basal keratinocyte proliferation and repair [33].

In BCC, EGFR contributes to tumorigenesis by interacting with the Hedgehog (HH)/GLI signaling axis, thereby enhancing oncogenic signaling via the RAS/RAF/MEK/ERK/JUN cascade. Combined inhibition of EGFR and HH/GLI pathways has demonstrated a synergistic reduction in BCC cell growth, underscoring EGFR’s role as a co-activator and potential therapeutic target [34].

Immunohistochemically, EGFR is frequently overexpressed in BCC compared to normal skin. Elevated EGFR expression has been associated with increased recurrence rates, particularly in infiltrative subtypes and in tumors with positive surgical margins, suggesting a link between EGFR expression and more aggressive clinical behavior [35].

The expression of EGFR has also been investigated in DFs. In the study by Morgan et al., dermal EGFR expression was found to decrease progressively throughout the histological evolution of DF, while epithelial expression remained constant. This has led to the hypothesis that EGFR may contribute to the epithelial hyperplasia observed in DFs through paracrine signaling. However, despite its biological involvement, EGFR expression is not considered a reliable marker for distinguishing BCC from basaloid epithelial proliferations overlying dermatofibromas [9].

Given the significant morphological and immunohistochemical overlap between superficial BCC and basaloid epithelial proliferations overlying DFs, there is an increasing need for more precise and objective diagnostic tools. In this context, molecular techniques have emerged as valuable adjuncts in the diagnostic algorithm, offering insights into the genetic and signaling landscapes of cutaneous tumors.

Among these methods, chromogenic in situ hybridization (CISH) for RNA transcripts has shown promise by enabling the visualization of gene expression within the native tissue architecture. One of the most widely studied molecular targets is GLI1, a transcription factor within the Hedgehog signaling pathway, which is constitutively activated in the vast majority of BCCs. GLI1 RNA CISH has demonstrated high sensitivity and specificity for BCC, even in histologically ambiguous or metastatic lesions, and has proven useful in distinguishing BCC from a broad spectrum of non-follicular epithelial neoplasms [36].

However, despite its diagnostic value, molecular profiling has important limitations. A major drawback is the lack of specificity of certain markers, particularly in differentiating BCC from benign follicular tumors, many of which also exhibit Hedgehog pathway activation. This shared molecular signature likely reflects a common developmental origin and complicates the genetic distinction between neoplastic and reactive basaloid proliferations. Furthermore, the cost, technical demands, and limited accessibility of molecular tools may restrict their routine implementation in standard dermatopathology practice.

## 5. Conclusions

BCH over DFs represents a benign yet diagnostically challenging phenomenon that can closely mimic superficial BCC. Our analysis, together with previous reports, demonstrates that conventional histology alone is insufficient to reliably distinguish these two entities, as both may share peripheral palisading, basaloid lobules, and peritumoral clefting. Immunohistochemical markers—including BerEP4, Ki-67, CK20, and others such as metallothionein, podoplanin, and EGFR—add little discriminatory power, while molecular approaches such as GLI1 RNA CISH, though promising, remain limited by issues of specificity, cost, and accessibility.

Despite these significant technical challenges, clinical evidence remains unequivocal: no recurrences, metastases, or locally aggressive growth patterns have ever been reported in DF-associated BCH or in other benign lesions that exhibit basaloid proliferations. This consistent benign course, repeatedly confirmed in the literature, stands in stark contrast to the well-documented progressive potential of BCC [37]. As such, while BCH may cause diagnostic uncertainty in daily practice, it does not appear to carry prognostic relevance for patients, and overinterpretation may lead only to unnecessary interventions.

Beyond its limited clinical impact, the true significance of BCH may lie in its biological implications. By displaying the cytological and architectural hallmarks of carcinoma without acquiring invasive behavior, basaloid proliferations provide a unique natural model for studying the earliest stages of tumorigenesis. Their investigation may help clarify the molecular events that precede malignant transformation, the stromal and epithelial interactions that constrain progression, and the mechanisms that determine why certain proliferative processes remain confined while others evolve into overt carcinoma. This experimental potential underscores the value of BCH not as a prognostic marker, but as a window into the fundamental biology of cancer initiation.

## Figures and Tables

**Table 1:** Histological Characteristics and IHC-Markers (Ber-Ep, Ki-67, CK-20).

Sample Nr	Lobules	Basaloid Cells	Palisade	Clefting	BerEp4	Ki67	CK20
**1**	1	1	1	0	pos	+	−
**2**	1	1	1	0	pos	++	+
**3**	1	1	1	1	pos (weak)	+++	−
**4**	1	1	1	1	pos	++	+
**5**	1	0	1	1	pos	++	−
**6**	1	1	1	0	pos	+++	+
**7**	1	1	1	1	pos	+	+
**8**	1	1	1	0	pos	++	−
**9**	1	1	1	1	pos	+++	−
**10**	1	1	1	0	pos (weak)	++	+
**11**	1	1	1	1	pos	++	+
**12**	1	1	1	0	pos	++	−
**13**	1	1	1	0	pos	++	+
**14**	1	1	1	1	pos	++	−
**15**	1	0	1	0	pos	++	+
**16**	1	1	1	1	pos (focal)	+	+
**17**	1	1	1	0	pos	++	−
**18**	1	1	1	0	pos	++	−
**19**	1	1	1	1	pos (focal)	++	−
**20**	1	1	1	0	pos (focal)	++	+
**21**	1	1	1	0	pos	++	+
**22**	1	1	1	0	pos (focal)	++	+
**23**	1	1	1	0	pos	++	−
**24**	1	1	1	0	pos	+++	−
**25**	1	1	1	0	pos	++	−
**26**	1	1	1	0	pos (focal)	nd	nd
**27**	1	1	1	0	pos (focal)	nd	nd
**28**	1	0	1	0	pos (focal)	nd	nd
**29**	1	1	1	0	pos	nd	nd
**30**	1	1	1	0	pos (focal)	nd	nd
**31**	1	1	1	0	pos	nd	nd
**32**	1	1	1	0	neg	nd	nd
**33**	1	1	1	0	neg	nd	nd
**34**	1	1	1	0	neg	nd	nd
**35**	1	1	1	0	neg	nd	nd
**36**	1	1	1	0	neg	nd	nd
**37**	1	1	1	0	neg	nd	nd
**38**	1	1	1	0	neg	nd	nd
**39**	1	1	1	0	neg	nd	nd
**40**	1	1	1	1	neg	nd	nd
**41**	1	1	1	0	neg	nd	nd
**42**	1	1	1	0	neg	nd	nd
**43**	1	1	1	0	neg	nd	nd

Abbreviations: 1 = present; 0 = absent; nd = not done; pos = positive; pos (weak) = weak positivity; pos (focal) = focal positivity; neg = negative; +/++/+++ = low, moderate, strong positivity; − = negative.

## Data Availability

The data presented in this study are available from the corresponding author upon reasonable request, in accordance with privacy and ethical restrictions.

## Abbreviations

The following abbreviations are used in this manuscript:

BCC	Basal Cell Carcinoma
BCH	Basaloid Cell Hyperplasia
CISH	Chromogenic In Situ Hybridization
CK20	Cytokeratin 20
DF	Dermatofibroma
EGF	Epidermal Growth Factor
EGFR	Epidermal Growth Factor Receptor
HE	Hematoxylin and Eosin
IHC	Immunohistochemistry
MT	Metallothionein
SCC	Squamous Cell Carcinoma
SCF	Stem Cell Factor
